# Endothelial dysfunction triggers acute respiratory distress syndrome in patients with sepsis: a narrative review

**DOI:** 10.3389/fmed.2023.1203827

**Published:** 2023-06-02

**Authors:** Rachael Cusack, Lieuwe D. Bos, Pedro Povoa, Ignacio Martin-Loeches

**Affiliations:** ^1^Department of Intensive Care, St. James’s Hospital, Dublin, Ireland; ^2^School of Medicine, Trinity College Dublin, Dublin, Ireland; ^3^Intensive Care, Amsterdam UMC Location AMC, University of Amsterdam, Amsterdam, Netherlands; ^4^NOVA Medical School, CHRC, New University of Lisbon, Lisbon, Portugal; ^5^Center for Clinical Epidemiology and Research Unit of Clinical Epidemiology, OUH Odense University Hospital, Odense, Denmark; ^6^Department of Intensive Care, Hospital de São Francisco Xavier, CHLO, Lisbon, Portugal

**Keywords:** acute respiratory distress syndrome, endothelial dysfunction, sub-phenotypes, microcirculation, sepsis, intensive care

## Abstract

Acute respiratory distress syndrome (ARDS) is a severe organ failure occurring mainly in critically ill patients as a result of different types of insults such as sepsis, trauma or aspiration. Sepsis is the main cause of ARDS, and it contributes to a high mortality and resources consumption both in hospital setting and in the community. ARDS develops mainly an acute respiratory failure with severe and often refractory hypoxemia. ARDS also has long term implications and sequelae. Endothelial damage plays an important role in the pathogenesis of ARDS. Understanding the mechanisms of ARDS presents opportunities for novel diagnostic and therapeutic targets. Biochemical signals can be used in concert to identify and classify patients into ARDS phenotypes allowing earlier effective treatment with personalised therapies. This is a narrative review where we aimed to flesh out the pathogenetic mechanisms and heterogeneity of ARDS. We examine the links between endothelium damage and its contribution to organ failure. We have also investigated future strategies for treatment with a special emphasis in endothelial damage.

## Introduction

The acute respiratory distress syndrome (ARDS) is a complex and varied syndrome that is characterized by severe and often refractory hypoxemia, resulting in a high morbidity and mortality rate (1). ARDS is caused by a local injury to the alveolar capillary membrane due to endothelial dysfunction, alveolar injury, or both. The most accurate estimate is that around 10% of invasively ventilated patients fulfil the criteria for ARDS (2). The current and most widely used definition requires an acute onset, radiographic bilateral infiltrates consistent with pulmonary (not cardiogenic reason) oedema and severe hypoxemia despite 5 cmH2O of positive end-expiratory pressure (PEEP) – so known as the Berlin criteria (3). This condition affects between 17 and 20 people per 100,000 annually, which represents nearly 5% of mechanically ventilated patients (4).

ARDS definition has been modified over the years and new ongoing definitions are being developed. Sub-phenotypes of ARDS have also been identified based on plasma biomarkers of inflammatory host response, endothelial dysfunction, and coagulopathy (5–7). Around 1/3 of patients with ARDS present with a hyper-inflammatory sub-phenotype, while the other 2/3 presents with a hypo-inflammatory sub-phenotype (8). While pulmonary endothelial dysfunction is frequently overlooked in ARDS pathophysiology, pre-clinical models have established that endothelial dysfunction can be responsible for development of pulmonary oedema (9). It is plausible to hypothesize that a subset of patients has an endothelial-driven injury sub-phenotype. This paper aims to summarize the pathophysiological links between endothelial dysfunction and injury and the development of ARDS. It will not only focus on biomarkers measurable in the systemic circulation but also on the analysis of local biochemical changes. Furthermore, it will explore the heterogeneity of these processes in patients with ARDS and review the available interventions to target the endothelium in ARDS.

## Methods

For this review a literature search was conducted on PubMed/MEDLINE, Embase and Google Scholar database using combinations of keywords; “acute respiratory distress syndrome,” “endothelium,” “endothelial dysfunction,” “heterogeneity” and “microcirculation.” Published articles focusing on the role, implications and treatment of endothelial dysfunction in ARDS were included. Relevant articles referenced within included reports were also accessed. We identified case studies, case series, observational studies, randomised controlled trials and review articles.

### Pathophysiology

ARDS is characterized by acute onset of hypoxemia, bilateral pulmonary infiltrates on chest imaging, and respiratory failure requiring mechanical ventilation (3). There are two main types of ARDS: direct and indirect. Direct ARDS refers to cases where the lung injury is caused by a direct insult to the lung tissue itself, such as pneumonia or aspiration (10). In direct ARDS, there is damage to the alveolar epithelial and endothelial cells, which can lead to the accumulation of fluid in the lungs, impaired gas exchange, and decreased lung compliance (11). Indirect ARDS, on the other hand, refers to cases where the lung injury is caused by an indirect insult, such as sepsis or trauma. In indirect ARDS, the lung injury is thought to be caused by an inflammatory response that is triggered by the systemic insult (12). This can lead to the activation of various immune cells and the release of inflammatory mediators, which can damage the pulmonary endothelium and alveolar epithelium, resulting in lung injury and respiratory failure.

The difference between pulmonary and extrapulmonary ARDS has been acknowledged for nearly 2 decades (13). The endothelium is primarily distorted following an extrapulmonary insult, with damage due to the action of inflammatory mediators in the systemic circulation that increase vascular permeability and oedema resulting in microcirculation congestion. As opposed to direct pulmonary insults, that activate alveolar macrophages and neutrophils, increasing IL-6 and altered type I and type II cells in bronchoalveolar lavage samples, leading to intrapulmonary inflammation and increased extracellular matrix remodelling (14). A recent prospective observational study of airspace fluid from 153 mechanically ventilated patients found increased glycosaminoglycan shedding in patients with direct lung injury aetiology for ARDS (15). This experiment showed a link between epithelial layer shedding and reduced surfactant, a pathological mechanism in ARDS that was also increased by duration of mechanical ventilation. Using this distinction and differentiating patients by source of ARDS or degree of endothelial involvement could be helpful in both predictive and prognostic enrichment of future studies.

#### Endothelial glycocalyx

The pulmonary endothelium consists of a single layer of mesenchyme-derived and non-fenestrated endothelial cells. The luminal surface of blood vessels is lined with endothelial cells which are in turn covered by a jelly-like layer of glycocalyx, made up of proteoglycans and glycosaminoglycans separating the intravascular compartment from the interstitium (16). The glycocalyx is important in maintaining the endothelial integrity and is implicated in coagulation, cell signalling and inflammation (17). It is composed of carbohydrate-like glycosaminoglycans, heparan sulfate, hyaluronic acid (HA) and syndecans (Table 1). The syndecan family of transmembrane proteoglycans consists of four main types (SDC1-4), and their expression levels can vary between different tissues and organs (18). Syndecan-1 (SDC1) is primarily expressed on the surface of epithelial cells and is involved in a variety of cellular functions (19–21). It has been implicated in mechanosensation, or the ability of cells to sense and respond to physical forces in their environment (22). SDC1 also plays a role in vascular permeability, or the ability of substances to pass through blood vessel walls, and has been shown to promote leukocyte adhesion, which is the attachment of white blood cells to the walls of blood vessels (22).

**Table 1:** Endothelial markers and the associations with ARDS.

Endothelial markers	Associations with ARDS
Syndecan 1	Negative correlation with PaO2/FiO2 Positive correlation with need for intubation Higher SOFA score & mortality Increased in cases of influenza A with ARDS Indirect ARDS/Extrapulmonary sepsis & ARDS Increased in non-survivors
Hyaluronan	Higher concentrations associated with ARDS
Soluble thrombomodulin	Increases with severity of ARDS Increased in non-survivors
Angiopoeitin 2	Independent predictor of mortality in ARDS Associated with infective source ARDS
Intercellular adhesion molecule	Expressed after interaction with bacteria Associated with protein rich pulmonary oedema
Selectins	Associated with ARDS severity & mortality Protein rich pulmonary oedema
Heparan sulfate	Indirect ARDS Increased with increasing fluid transfusion
Hyaluronic acid	Direct ARDS

SOFA, sequential organ failure assessment score; ARDS, acute respiratory disease syndrome.

In addition to these functions, SDC1 has been implicated in a variety of other biological processes, including cell migration, differentiation, and proliferation. It is also involved in wound healing and tissue regeneration and has been shown to interact with a variety of signalling molecules, including growth factors and extracellular matrix proteins.

##### Pulmonary endothelium

The function of the lungs in gas exchange means the endothelium here is unique from endothelium in other parts of the body. The lungs are a low-pressure system where the entire blood volume passes through. Hypoxic pulmonary vasoconstriction (HPV) is a physiological response of the lungs to low oxygen levels in the alveoli (the tiny air sacs where gas exchange occurs). This response involves the constriction of blood vessels in the lungs, which helps to redirect blood flow to areas of the lungs with better oxygenation and improve the efficiency of gas exchange. While the systemic circulation vasodilates in response to low oxygen signals, the pulmonary vessels constrict to redirect blood flow to better aerated alveoli. This response is more pronounced as the diameter of the vessels decreases (23). The pulmonary endothelium is highly metabolically active and involved in maintaining vascular tone through nitric oxide, prostacyclin, serotonin and endothelin production (24).

**Figure 1:**
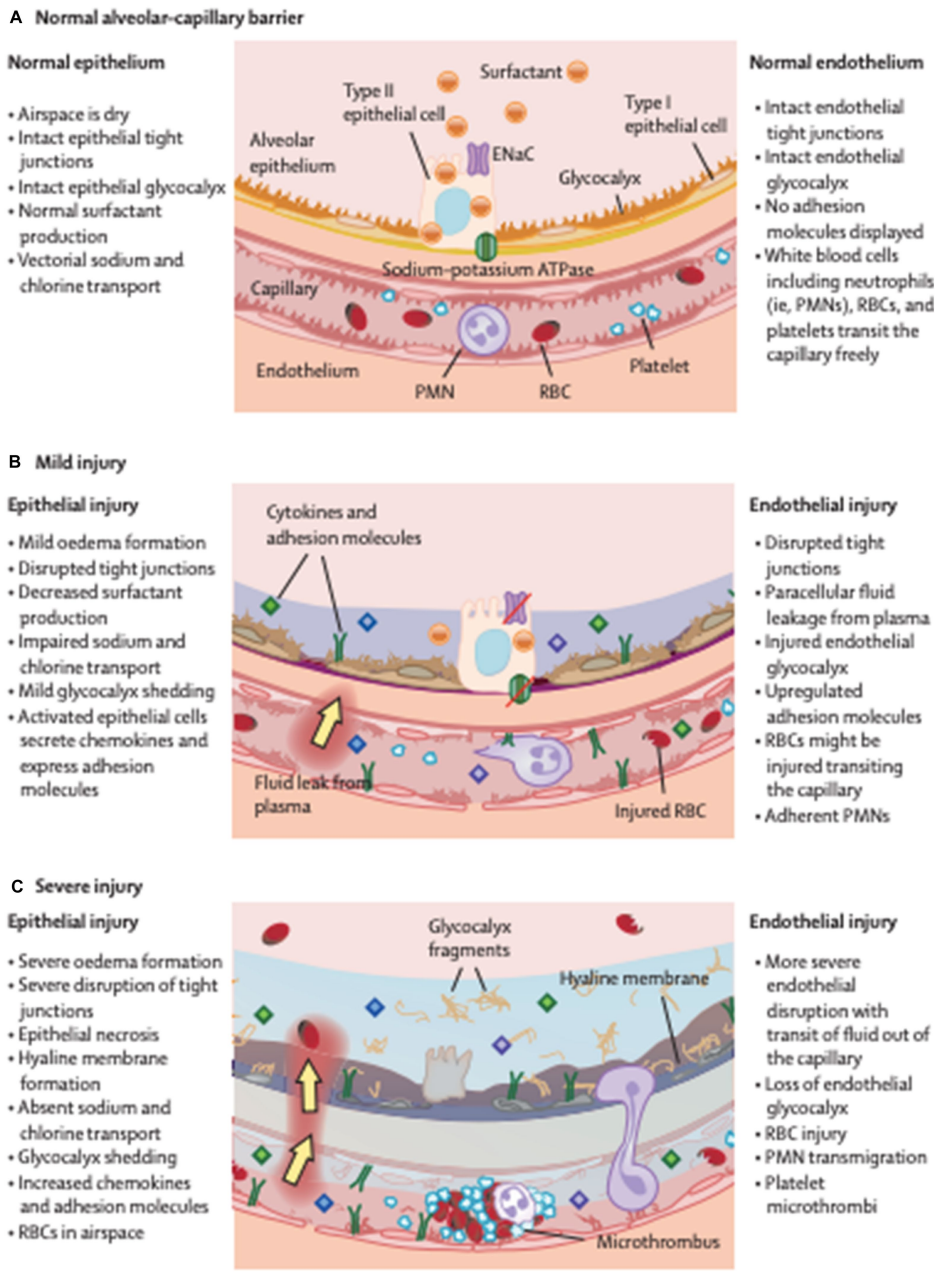
A barrier called between the alveolus and capillary prevents the formation of oedema in health **(A)**. This deteriorates in mild **(B)** and further in severe injury **(C)**, becoming more permeable leading to the development of protein rish oedema in the lungs. ENaC, epithelial sodium channel; PMN, polymorphonuclear cells; RBC, red blood cell. Reproduced with permission from The Lancet.

One of the main functions of the pulmonary vascular endothelium is to preserve the airspaces against vascular fluid. The permeability of this membrane is concerned in several disease states, indicative of the health of different organs. Permeability leads to trans-endothelial movement of fluid and cells into airspaces (Figure 1).

The lungs are also an important site of immunological defence, so the endothelium is specialised at vascular branch points, which act as filters to trap and transport potential antigens to local lymph nodes (25). The immune response recruits inflammatory cells to the lung where adjacent cells respond by releasing inflammatory substances such as arachidonate, ATP and reactive oxygen species which damage the endothelium (26–28). ARDS precipitants such as gastric acid and mechanical stress increase mitochondrial transcription of leukocyte adhesion molecules (28). ARDS generating conditions increase the concentration of mitochondrial cytosolic calcium which leads to nuclear factor kappa B (NFκB) production through a hydrogen peroxide pathway. This activation leads to production of E-selectin and P-selectin, important inflammatory and adhesion molecules in the lung.

##### Alveolar glycocalyx

**Figure 2:**
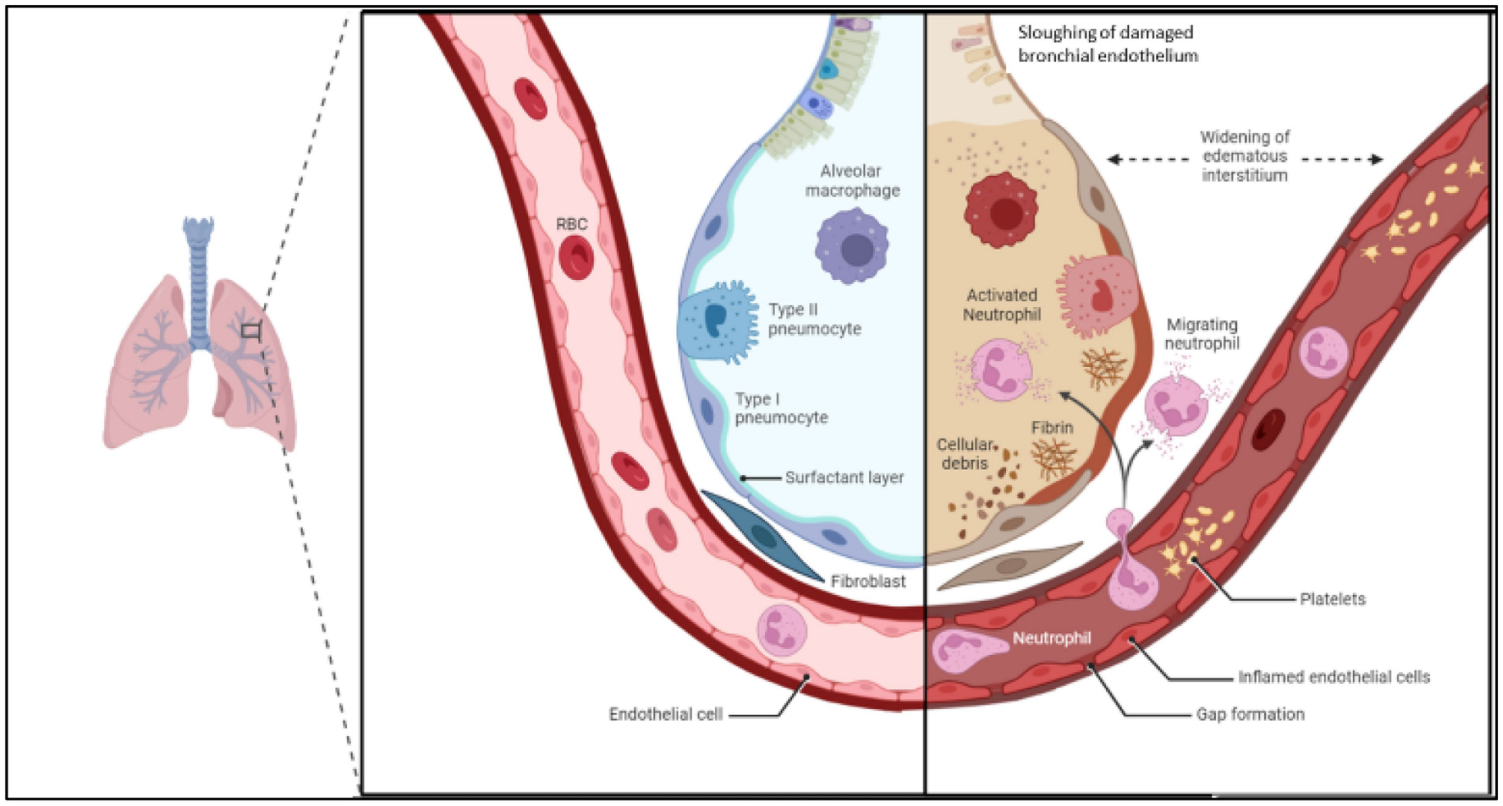
Demonstrating changes in endothelial permeability and alveolus in acute respiratory distress syndrome. Inflammatory cascade signals breakdown endothelial cell adhesion molecules, widening space between endothelial cells. Increased expression of leukocyte adhesion molecules increases leukocyte rolling and migration across the endothelial barrier, where cytokine release, reactive oxygen species and damaging enzymes increase the inflammatory feedback loop. This leads to oedema and sloughing off of bronchial endothelium, which reduced the gas exchange ability of the alveolus. Image adapted from Biorender.

The alveolar glycocalyx is a layer of glycoproteins and proteoglycans that covers the surface of the alveolar epithelium, which is the tissue that lines the alveoli in the lungs (29). The glycocalyx is a part of the extracellular matrix of the alveoli and plays an important role in maintaining the integrity and function of the alveolar barrier (30). The alveolar glycocalyx is involved in regulating the exchange of fluids, electrolytes, and other molecules across the alveolar epithelium, which is essential for normal lung function (Figure 1). It also helps to protect the alveolar epithelium from damage caused by mechanical stress and inflammation. Recent studies have suggested that the alveolar glycocalyx may play a role in the pathogenesis of ARDS (15, 30, 31). Damage to the glycocalyx has been shown to increase the permeability of the alveolar barrier and contribute to the development of pulmonary oedema (Figure 2). Animal models of inhaled pulmonary insults lead to alveolar epithelial glycocalyx breakdown, loss of surfactant and reduced lung function (11). Studies have shown that direct pulmonary insults lead to epithelial glycocalyx breakdown and are correlated to PaO2/FiO2 and outcomes (duration of mechanical ventilation) in ARDS patients (15). This distinction between the pathogenesis of direct and indirect lung injury in ARDS could lead to new diagnostic and treatment targets (32).

##### Endothelial glycocalyx destruction and ARDS

The destruction of the endothelial glycocalyx has been associated with increased vascular permeability, leukocyte adhesion, and inflammation in the lungs (33, 34). Moreover, clinical studies have demonstrated that patients with ARDS have increased levels of glycocalyx components in their circulation, suggesting that glycocalyx damage may be a feature of the disease (35–37). Although the exact mechanisms underlying glycocalyx destruction in ARDS are not fully understood, it is thought to be related to a combination of factors, including oxidative stress, inflammation, and mechanical stress.

Animal models have been used to demonstrate the relationship between endotoxemia and loss of endothelial thickness (38–41). LPS injection causes increased pulmonary capillary permeability with endothelial leak that recovers over 96 h (40).

##### Disrupted tight junctions

Tight junctions between endothelial cells maintain barrier integrity, which is dependent on vascular endothelial cadherin (VE-cadherin) (42). Endothelial permeability is regulated by adherens junctions and tight junctions, particularly on the venular side where there are molecular targets for inflammatory mediators such as tumour necrosis factor (TNF), interleukin-1 (IL-1) and vascular endothelial growth factor (VEGF) (42, 43). Endothelial leak is promoted by intracellular sequestration of VE-cadherin which is initiated by mediators such as thrombin and VEGF (44). Inflammatory mediators thrombin and VEGF stimulate dismantling of adherens and tight junctions in a calcium dependent manner by phosphorylating VE-cadherin, leading to actin chain contraction and increased intercellular permeability (45).

##### Angiopoeitin-2

Angiopoeitin 2 (Ang-2) is a marker of endothelial dysfunction and has therefore been studied extensively in patients with or at risk for ARDS. Ang-2 works with Tie-2 and in opposition to Ang-1 to control permeability throughout the vascular network. A retrospective analysis of 931 patients from the NHLBI ARDS Network randomised controlled trial (RCT) fluid liberal vs. fluid conservative management strategy in ALI (FACTT) trial found that Ang-2 was an independent predictor of mortality in ARDS as well as being statistically significantly associated with infectious aetiology of acute lung injury (ALI) (37). A meta-analysis of prospective cohort studies focussing on Ang-2 and risk of mortality in ARDS found increased odds of death in patients with a raised baseline Ang-2 level (OR 1.56, 95% CI 1.3–1.89) (46). In patients with infection related ARDS, Ang-2 levels higher on day 3 than on day 0 was independently associated with death at an OR 2.29 (95% CI 1.54–3.43, *p* < 0.001). Ang-2 levels on day 3 were associated with mortality in infection related ARDS (OR 1.64, 95% Ci 1.32–2.03, p < 0.001) as well as in non-infection related ARDS (OR 2.03, 95% CI 1.31–3.16, *p* = 0.002). Patients in the fluid conservative group had a 13% greater decline in Ang-2 over the 3 days (*p* = 0.005) and this effect was more in those patients who were not in shock and experienced a 19.9% fall in Ang-2 levels (37).

Using Mendelian randomisation a causal link between Ang-2 and ARDS was investigated (47). In this study the authors found that patients with European ancestry who had sepsis were more likely to develop ARDS if they expressed the ANGPT2 genetic variant. Five ANGPT2 gene variants had associations with ARDS in sepsis patients with European ancestry. This not only describes a potential subset of patients but also highlights how genetic data could be used to classify and phenotype cohorts in ARDS.

A study of single nucleotide polymorphisms (SNPs) in 225 ARDS patients found an association with FLT1, which encodes a VEGF receptor (48). The authors proposed this as a potential mechanistic link between endothelial damage and ARDS. Forty-nine percent of patients in the cohort had positive blood cultures with Gram-negative bacteria identified and 49% had direct pulmonary injury resulting in ARDS (48). Utilizing omics data represents a promising approach for elucidating the underlying mechanisms of intricate pathologies, such as ARDS.

Intercellular adhesion molecule (ICAM) is a cell surface glycoprotein that plays an important role in leukocyte adhesion and trans-endothelial migration during inflammation (49). ICAM expression on endothelial cells can induce actin stress fibre formation, which is a cytoskeletal structure composed of contractile actin filaments that are important for maintaining cell shape and motility (50). ICAM expression can also increase endothelial permeability through a positive feedback loop involving the activation of intracellular signalling pathways, such as the RhoA/ROCK pathway. The formation of actin stress fibres induced by ICAM expression can activate RhoA/ROCK signalling, which in turn leads to the assembly of actin-myosin contractile structures and increased endothelial permeability (51). This positive feedback loop between ICAM expression, actin stress fibre formation, and increased permeability is thought to play an important role in the pathogenesis of various inflammatory disorders, including acute lung injury and sepsis. In these conditions, increased ICAM expression and activation of RhoA/ROCK signalling can lead to a breakdown of the endothelial barrier, resulting in the leakage of fluid and cells into the interstitial space and impaired organ function (49).

##### Intracapillary red cell injury

Alveolar red blood cells and pulmonary haemorrhage are also features of ARDS. RBC rheology in sepsis is affected by several factors, including increased nitric oxide release affecting membrane deformability as well as increased aggregation and increased viscosity (52). Altered membrane deformability can lead to haemolysis and the release of cell free haemoglobin, which travels into alveoli and causes damage (53). This was proposed as a mechanism causing transfusion related acute lung injury (TRALI) however, two RCTs have disputed this mechanism, showing no benefit of fresh RBCs in overall survival of critically ill patients (54, 55). Cell-free haemoglobin precipitates release of reactive oxygen species (ROS) which impairs the endothelial integrity, leading to vasculopathy (56, 57). Large cohort studies have been employed to investigate the influence of cell free haemoglobin and pulmonary hypertension in sickle cell anaemia (56, 58, 59) RBC haemolysis products can be considered danger-associated molecular patterns (DAMPs) that could cause lung injury by damaging the endothelium (60).

#### Heterogeneity in endothelial dysfunction in patients with ARDS

Patients with a hyper-inflammatory ARDS sub-phenotype also have higher plasma concentrations of biomarkers indicative of endothelial dysfunction (Table 2) (61). They frequently experience multi-organ failure and shock, all consistent with profound endothelial dysfunction. Latent class analysis has been used to identify hyper- and hypo-inflammatory sub-phenotypes of ARDS, which are consistent across a number of large randomised controlled trials, with specific biological and clinical characteristics affecting treatment response and prognosis (8, 62–64). Pathophysiological mechanisms affecting the endothelial barrier function are unlikely to be activated simultaneously in all patients who develop ARDS. When comparing patients with pulmonary and non-pulmonary causes for ARDS, the latter has higher levels of circulating biomarkers indicative of endothelial dysfunction (such as Ang2, IL-8 and vWF) (12, 34). Combined with the observation that patients with non-pulmonary sepsis frequently develop multi-organ failure and that in all of these organs endothelial dysfunction may contribute to failure of the organ, it has been suggested that endothelial dysfunction plays a more important role in this subset of patients (36).

**Table 2:** Sub-phenotypes of ARDS, biomarkers and features.

Sub-phenotype	Biomarkers	Features
Hyper-inflammatory	Higher concentrations IL-6, IL-8, TNF receptor-1, and PAI-1, ICAM, vWF, Surfactant protein-D	More sepsis Higher heart rate Higher total minute ventilation Lower SBP Lower HCO3^−^ Lower protein C X3 higher vasopressor at baseline Higher mortality
Hypo-inflammatory	Lower concentrations IL-6, IL-8, PAI-1, ICAM, vWF	More trauma related More organ failure free days More ventilator free days Lower mortality
Pulmonary	Alveolar macrophages, neutrophils IL-6 Type I & type II cells Reduced surfactant GAG shedding	Direct pulmonary injury
Extra-pulmonary	Increased Ang-2, IL-8, vWF Heparan sulfate SDC-1	Non-pulmonary sepsis Multi-organ failure

IL-6, interleukin 6; IL-8, interleukin 8; TNF, tumour necrosis factor; PAI, plasminogen activator inhibitor; ICAM, intercellular adhesion molecule; vWF, von Willebrand factor; SPD, surfactant protein D; SBP, systolic blood pressure; HCO3, bicarbonate; GAG, glycosaminoglycan; Ang-2, angiopoeitin 2; SDC-1, syndecan-1.

The Fluid and Catheter Treatment Trial (FACTT) identified 2 definite sub-phenotypes of ARDS that responded differently to fluid management (62). The authors randomly assigned ARDS patients to receive either conservative or liberal fluid management strategy. They found that those with higher inflammatory markers and hypotension had 40% mortality when treated with a conservative strategy and 50% mortality in the liberal group. However if a patient was not in this hyperinflammatory group they had a 26% mortality in fluid-conservative and 18% mortality if liberally treated with fluid (62). This suggests that sub-phenotyping patients according to clinical markers can influence treatment decisions. Potentially biochemical or omics driven sub-typing could impact treatment as well.

##### COVID-19 and pulmonary endothelium

The pulmonary endothelium plays an important role in COVID-19 related lung injury by blood flow regulation, maintaining vascular integrity, and preventing the extravasation of fluid and cells into the lung tissue (65). In COVID-19, the virus can infect pulmonary endothelial cells through the ACE2 receptor, which is expressed on the surface of these cells (66). The viral infection can lead to endothelial dysfunction, inflammation, and increased vascular permeability, which may contribute to the development of severe respiratory symptoms and organ dysfunction in COVID-19 patients (67–69). The endothelial dysfunction can also lead to the pro-inflammatory and pro-thrombotic state in the pulmonary endothelium with formation of microthrombi further exacerbating tissue damage (70).

#### Treatments targeting endothelial dysfunction

Despite great efforts there has been equivocal results in the search for pharmacologic agents to treat ARDS, a Cochrane meta-analysis could find no benefit of any recently investigated therapies (71). Research around therapeutic options in ARDS has focused on inhibiting the damaging aspects of the immune response and endothelial degradation. Targeting the endothelium and it’s regeneration would offer an important goal for predictive enhancement of future trials. Those with a higher proportion of endothelial damage involvement (non-pulmonary sepsis) might benefit more from drugs targeting the endothelium.

##### Therapies targeting glycocalyx

Fluid therapy for patients with sepsis is the standard of care worldwide (72, 73). Excessive fluid resuscitation and hyper-oncotic solutions have been shown to increase damage to the glycocalyx (74). The FINNAKI trial showed that fluid administration is correlated to amount of fluid transfused and non-survivors had more SDC-1 and thrombomodulin circulating than survivors (75). Glycocalyx damage assessed by peripheral heparan sulfate levels was also shown to correlate with each litre of intravenous fluids administered (74). Excess fluid administration is associated with poorer outcomes in sepsis (76–78). Albumin also preserves the glycocalyx and reduced leukocyte adhesion and SDC-1 concentrations (79). A recent review found that plasma and albumin were superior to crystalloids and colloids in preserving the glycocalyx (Table 3) (84). Conservative fluid resuscitation strategies in ARDS patients aim to prevent pulmonary oedema and may act by preventing glycocalyx breakdown and leakage.

**Table 3:** ARDS therapies and benefits in ARDS (39, 62, 79–81), (82, p. 21), (83).

Therapeutics	Benefits in ARDS
Conservative fluid therapy	Reduced glycocalyx damage measured by heparan sulfate concentration Prevent pulmonary oedema
Albumin	Reduced leukocyte adhesion and SDC-1 concentration
Recombinant thrombomodulin	Less endothelial disruption, oedema, inflammation Enhance glycocalyx synthesis
GSK2586881 (Recombinant human angiotensin converting enzyme type 2)	Anti-inflammatory Benefits in COVID-19
Imatinib mesylate	Reduced mortality in COVID-19 ARDS Reverses endothelial dysfunction and improves immunomodulation
Mesenchymal stromal stem cells	Anti-inflammatory and endothelial restoration
Statin	Improved survival in ARDS hyper-inflammatory subphenotype

Recombinant thrombomodulin has also been investigated as a potential therapy to induce endothelial glycocalyx repair in ARDS. Thrombomodulin is a key component of the endothelial glycocalyx with anticoagulant effect, binding thrombin to generate activated protein C. By neutralizing high mobility group B1 (HMG-B1) released by necrotic cells it attenuates inflammation (85). Recombinant thrombomodulin has been investigated as a treatment for sepsis induced coagulopathy due to its effect on protein C (86). However, the SCARLET trial found no benefit at 28-days for patients with sepsis and DIC who received rhTM. A study investigating the possible effects of recombinant thrombomodulin on LPS-induced ARDS in mice administered recombinant thrombomodulin, which was associated with less capillary endothelial disruption and oedema than control (39). Genetic analysis suggested that recombinant thrombomodulin treatment may affect anti-inflammatory, cell proliferative and glycocalyx synthesis pathways and recombinant thrombomodulin might enhance glycocalyx synthesis. Survival of recombinant thrombomodulin treated mice was significantly higher than control mice after 48 h. Another study found that recombinant thrombomodulin might be a regulator of inflammation in the lung endothelium and protect against endothelial damage due to *Streptococcus pneumoniae* (87). The effect of recombinant thrombomodulin to reduce inflammation and possibly influence glycocalyx repair in ARDS warrants further investigation.

Neutrophil elastase, released from activated neutrophils in the immune response, damages pulmonary endothelium and is a factor contributing to ARDS (41). Sivelestat is a competitive inhibitor of neutrophil elastase, preventing pulmonary endothelial permeability but not affecting the immune response elsewhere (88). Though it has shown some efficacy in treating ALI, ARDS and SARS-CoV2,poorly designed, negative and small trials have hampered its adoption. Although some trials have shown a connection between PaO2/FiO2 and sivelestat therapy, there was no improvement in ventilation days or mortality (89–92). Retrospective studies have also had their validity questioned based on how they selected patients to show benefit in sepsis patients with disseminated intravascular coagulation and ARDS (93). Despite this controversy, the potential for a neutrophil elastase inhibitor to improve outcomes in ARDS is intriguing. A well designed randomised trial, enriched with subgroups of at risk patients could answer these questions in the future (94).

##### Therapies targeting angiopoietin system

The negative association of high Ang-2 with mortality in ARDS has also gained attention as a possible therapeutic target. Ang-2 is a product of the renin-angiotensin-aldosterone system (RAAS) that is responsible for blood pressure, vascular permeability, vasodilation and sodium absorption. GSK2586881 is a recombinant human angiotensin converting enzyme type 2 that was studied in ARDS and aimed to reduce levels of Ang-2, increasing Ang (1–7) thereby promoting anti-inflammatory and vasodilatory effects (95). The study was terminated early for failing to meet primary endpoints, though it has gained some attention for its potential treating COVID-19, there have been no further trials in ARDS, that we are aware of (80, 96). The involvement in of the RAAS in development of ARDS or patient’s failure to progress from mechanical ventilation could represent an important biologic marker for future prognostic enrichment (97, 98).

Imatinib mesylate is a small molecule therapy that was initially developed for cancer treatment with several targets including Abl, Arg (Abl-related gene), the stem-cell factor receptor (c-KIT) and platelet-derived growth factor receptor (PDGF-R) (99). Through its effect on Rac1 transmission and Arg, it improves endothelial cell barrier function and prevents vascular leak (100). Imatinib protects the endothelial barrier through inhibition of the ABL-2 tyrosine kinase, which is a key regulator of barrier function (100, 101). *In vitro* and mouse models have highlighted its possible differential effect in treating LPS-induced and ventilator induced lung injury models of ARDS (102). This raises an interesting question about the potential uses of the drug in homogenous populations of ARDS patients, depending on the inciting cause. A multicentre randomised placebo controlled trial of imatinib in COVID-19 disease found imatinib reduced mortality (14% placebo group vs. 8% imatinib group; adjusted hazard ratio 0.52, CI 0.26–1.05, *p* = 0.068) (103). Although the primary outcome of this trial, time to discontinuation of supplemental oxygen, did not reach statistical significance. Secondary analysis found that the reduction in mortality attributed to imatinib was completely mediated through immunomodulation and reversal of endothelial barrier dysfunction (81). Imatinib has shown some efficacy in treatment of pulmonary vaso-occlusive disease and post-chemotherapy fibrotic lung disease (104, 105). Case reports and series have emphasised imatinib’s usefulness treating various syndromes of endothelial leak, meaning it could have an important role in the future treatment of a subset of ARDS patients (82, 105, 106).

##### Alternative endothelial targeting therapies

Other treatments for ARDS targeting the endothelium have been sought out. Including old Chinese remedies such as Crocin, that may inhibit inflammation signalling as well as heparanase and matrix metalloproteinase 9 to preserve heparan sulfate and syndecan-4 in LPS induced ARDS (107). Therapies such as mesenchymal stromal stem cells have also been investigated for their anti-inflammatory and endothelial restorative abilities (83). Statins have also been investigated for their ability to reduce vascular leak in ARDS. HARP-2 and SAILS trials did not find any benefit of simvastatin or rosuvastatin in ARDS (108, 109). Analyses of the results of these trials did subsequently identify sub-phenotypes that may benefit from statin therapy. ARDS patients with hyperinflammatory sub-phenotypes had a higher survival rate with simvastatin compared to placebo (110).

## Future directions

Combining radiological, clinical and biochemical signals to accurately classify patients is key to delivering appropriate and timely care (111). Differential effects of non-pulmonary and pulmonary sepsis on the aetiology of ARDS provide opportunities for prognostic and predictive enrichment. Ang-2 and the renin-angiotensin-aldosterone system have been implicated as an important mediator in ARDS and the underlying structural lung damage, which could represent a target for predictive enrichment (112). Finding more targets such as these will depend on the application of multi-omics data and specific pathway enrichment studies (113). Until a single biomarker or point of care test can be prospectively tested, we can use large datasets to identify characteristic traits (114). Sub-classifying patients according to endothelial involvement or epithelial impact on disease could help develop treatments or diagnostic tests to catch at risk groups earlier. Identifying subtypes of disease and pathogenesis, predictive enrichment to reduce patient heterogeneity in trial design (115, 116). Genome studies, predictive pathway analysis and large repositories of omics data will help identify mechanisms and potential targets for treatment.

## Conclusion

Here we have discussed the mechanisms of endothelial dysfunction underlying sub-phenotypes of ARDS. Endothelial dysfunction in the pathogenesis of sepsis and ARDS is an important target for new diagnostic, prognostic and therapeutic targets in ICU. Identifying genetic and phenotypic disposition to more severe sub-phenotypes of critical illness will enhance the delivery of personalised medicine in the future.

## Author contributions

RC and IM-L developed the concept for the article. RC researched and wrote the article. LB and PP contributed figures and expertise. LB, PP, and IM-L reviewed and contributed to the final manuscript. All authors contributed to the article and approved the submitted version.

## Conflict of interest

The authors declare that the research was conducted in the absence of any commercial or financial relationships that could be construed as a potential conflict of interest.

## Publisher’s note

All claims expressed in this article are solely those of the authors and do not necessarily represent those of their affiliated organizations, or those of the publisher, the editors and the reviewers. Any product that may be evaluated in this article, or claim that may be made by its manufacturer, is not guaranteed or endorsed by the publisher.
